# Case Report: 21 Cases of Umbilical Hernia Repair Using a Laparoscopic Cephalic Approach Plus a Posterior Sheath and Extraperitoneal Approach

**DOI:** 10.3389/fsurg.2021.705469

**Published:** 2021-09-09

**Authors:** Kunjie Zhang, Mingfang Qin, Guoqian Ding

**Affiliations:** ^1^Department of General Surgery, The First Affiliated Hospital of Henan University of Traditional Chinese Medicine, Zhengzhou, China; ^2^The Third Department of General Surgery, Tianjin Nankai Hospital, Tianjin, China; ^3^Beijing Friendship Hospital, Capital Medical University, Beijing, China

**Keywords:** laparoscopic cephalic approach, posterior sheath, extraperitoneal approach, umbilical hernia, laparoscopic - methods

## Abstract

**Purpose:** In this study, a novel surgical technique was developed for umbilical hernias, in which a laparoscopic cephalic approach plus a posterior sheath and an extraperitoneal approach was employed. The aim of this study was to determine the results of this new technique.

**Methods:** From 2019 to 2020, 21 patients (81.8% men) with an umbilical hernia underwent a laparoscopic cephalic approach plus a posterior sheath and extraperitoneal approach, performed by two surgeons specializing in abdominal wall surgery, in two academic hospitals. Intraoperative and postoperative complications, operation time, blood loss, and hernia recurrence were assessed.

**Results:** Twenty-one cases of umbilical hernia were successfully completed. The size of the hernia ring was 1.5–3 cm^2^, with an average of 2.39 ± 0.47 cm^2^. The operation time was 120–240 min (average, 177.3 ± 42.15 min), and the blood loss volume was 30–40 ml (average, 33.73 ± 3.55 ml). The mean follow-up period was 6 months, and there were no short-term complications and no cases of recurrence.

**Conclusion:** A laparoscopic cephalic approach plus a posterior sheath and extraperitoneal approach is a safe alternative for the repair of an umbilical hernia. The intraoperative complication rate was low.

## Introduction

An umbilical hernia refers to an external abdominal hernia in which the contents of the abdominal cavity protrude from the weakened umbilical area (1). The umbilicus is located in the middle of the abdominal wall, which is the last part of the abdominal wall to close during embryonic development (2). The lack of adipose tissue in the umbilical area leads to the outermost skin, fascia, and peritoneum of the abdominal wall being directly connected together, making it the weakest part of the abdominal wall (3). The contents of the abdominal cavity protrude from this part to form an umbilical hernia. Obesity is the main cause of adult umbilical hernias due to increased abdominal pressure (4). The current treatment for umbilical hernias includes traditional open surgery, laparoscopic repairs, or even robotic repairs (1, 5–7). The entirely extraperitoneal technique for repair of inguinal hernia was first reported in 1992 by Dulucq (8) and has since become one of the gold standard procedures for the treatment of hernias in adults (9), such as umbilical hernias (10). We used a laparoscopic cephalic approach plus a posterior sheath and extraperitoneal approach to repair the umbilical hernias in this study, avoiding many complications caused by patch placements in the abdominal cavity, and achieved good clinical treatment results. The report is as follows.

## Materials and Methods

### General Information

There were 21 patients in this group, including 14 men and 7 women, aged 31–78 years, with an average age of 45.18 ± 13.58 years. The preoperative BMI of the patients was 24–35, with an average of 30.91 ± 4.55. All patients were clearly diagnosed with an umbilical hernia before surgery. The size of the hernia ring was 1.5–3 cm^2^, with an average of 2.39 ± 0.47 cm^2^. The period was from the first case on November 7, 2019, to the last case on November 11, 2020.

### Surgical Methods

**Figure 1:**
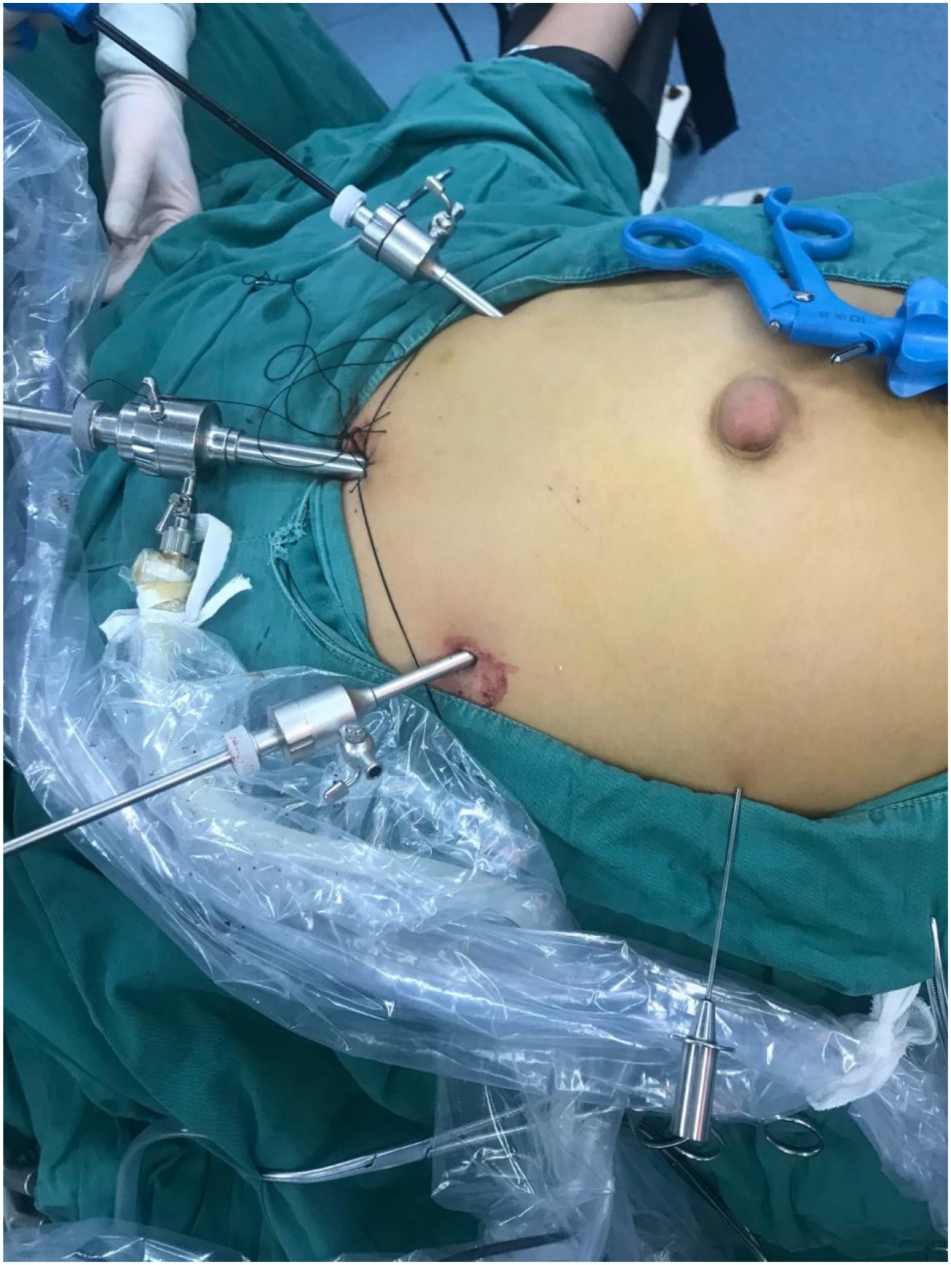
Punch position.

Endotracheal inhalation and intravenous combined general anesthesia were administered. The patient was placed in the supine position with the legs close together. The surgeon stands directly above the patient's head, and the first assistant stands on the left or right side of the patient's head. The punch position is shown in Figure 1.

A longitudinal incision was made about 3 cm under the xiphoid process, ~1.5 cm in length. The skin and subcutaneous tissue was cut first, then the anterior sheath was located; the anterior sheath was cut longitudinally, and 3–0 Medtronic absorbable sutures were applied using round needles and later smeared with paraffin oil. The left and right sides of the cut of the anterior sheath were sutured by continuous stitching and reserved for future use. The pull opened the rectus abdominis muscle, exposing the posterior sheath; the posterior sheath was cut, and, 3–0 Medtronic absorbable sutures were applied using round needles. The left and right sides of the incision of the sheath were sutured continuously, and then pulled for later use. The thin peritoneum and preperitoneal fascia were exposed.

A wet gauze was applied to carefully free the gap between the peritoneum and the posterior sheath. Care was taken to not break the peritoneum in order to maintain its integrity. Wet gauze was used to free an area of ~5 × 5 cm^2^, then the gauze was withdrawn, a 10 mm trocar was inserted along with a 10 mm lens, and the lens was used to perform a pushing action to free the preperitoneal space. The left or right side can be freed first by taking the puncture hole under the xiphoid process as a starting point, taking the midline as one side, and choosing the other side with an angle of 45°, ~5 cm in length, along with two other puncture holes on the left and right sides of the midline. We used gauze balls to free the extraperitoneal space, so one of the holes was 5 mm and the other was 10 mm, which is convenient for the gauze to enter and exit.

After the gap was released, hernia needles and non-absorbable sutures were used to close the hernia ring, place a common polypropylene patch in the extraperitoneal space, fix the patch with four needles, and place a drainage tube to routinely close the incision.

## Summary of the Cephalic Approach

### Discussion

The lens hole is set at about 3 cm below the xiphoid process, about 1 cm to the left or 1 cm to the right of the midline. This is to avoid the white line. After pulling the rectus abdominis, one should go straight to the posterior sheath. Otherwise, if the white line is entered, it will be difficult to find the right level.

When the anterior sheath is cut longitudinally through the lens hole, the length of the cut edges on both sides is ~1.5 cm. Johnson or Medtronic 3–0 absorbable sutures are used to suture the two cutting edges of the anterior sheath from top to bottom or from bottom to top. The margin should be ~1.5 mm, the needle pitch should be 1 mm; the end should be reserved and fixed with a vascular clamp. The same is done after the posterior sheath has been incised. In addition, when the posterior sheath is incised, it is not cut too deeply. One must gently cut the posterior sheath with the tip of the knife. Otherwise, if the peritoneum is cut too deeply and the peritoneum is cut, the cranial approach will fail because it is difficult to cut through the peritoneum. The peritoneum was free at the broken site and had moved into the extraperitoneal space.

At the center of the patch, stitch an absorbable suture to mark it. The purpose is to align the hernia ring of the umbilical hernia.

### After Patch Placement

#### Method 1

**Figure 2:**
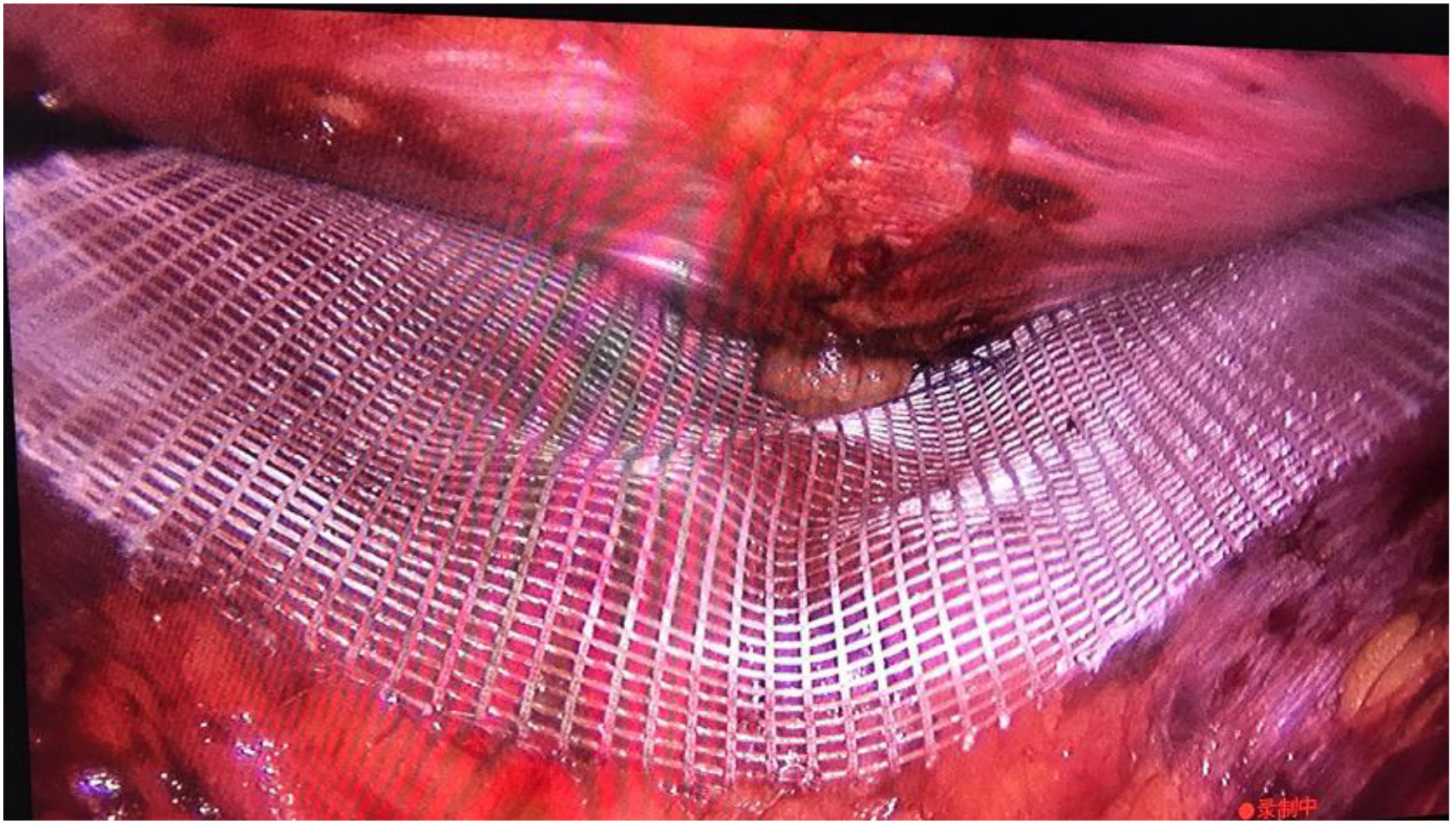
Method 1 of patch placement.

After inserting the patch, deflate, and press the hernia ring that is outside of the body. It can be seen that the hernia ring protrudes to the extraperitoneal space (the gap in which we operate) under an external force. When it is aligned in a vertical state, this indicates that the hernia ring faces the patch, ideally in the center of the circle. The patch is centered on the suture marking line and is equilateral around it. This is the ideal situation, as shown in Figure 2.

#### Method 2

**Figure 3:**
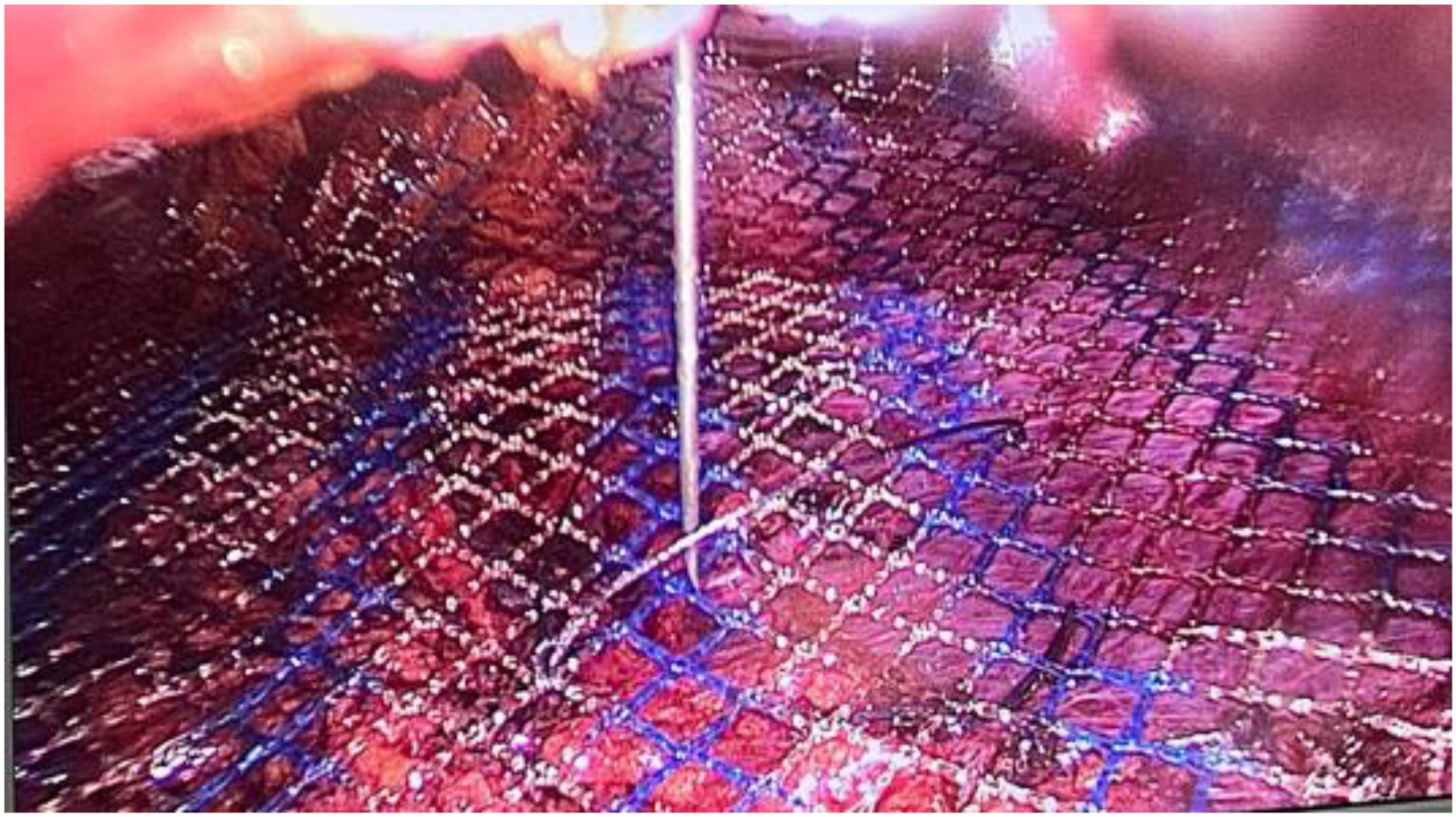
Method 2 of patch placement.

Use a 10 ml syringe needle instead of pressing the hernia ring. The needle vertically enters the extraperitoneal space, the pneumoperitoneum is slightly deflated, and the tip of the needle is aligned with the mark of the absorbable line, which has the same effect (see Figure 3).

Whether the patch is round or quadrilateral, the four corners of the patch need to be fixed with four needles to the peritoneum or extraperitoneal fascia, so that the patch will not easily move or shift (11). Only four needles need to be fixed, and there is no need to fix them too much.

After the operation, try not to enter the abdominal cavity for checks or visualization. It is necessary to cut the peritoneum into the abdominal cavity in order to perform inspections. In obese patients in particular, the peritoneum is very deep and difficult to suture when inspections are performed. Peritoneum sutures are not ideal, especially if the hole is close to the patch, which may cause the intestinal tube to enter the extraperitoneal space and cause an intestinal obstruction. Additionally, the adhesion of the patch between the intestine, omentum, and the extraperitoneal space may cause related complications.

If the peritoneum is accidentally broken during the process of freeing the peritoneum, one can use a pneumoperitoneum to exhaust gas. If necessary, one can use two insufflation needles to exhaust gas.

Both operation holes should be 5 mm or a 10 mm trocar can be used for one of the operation holes. We are personally accustomed to using gauze to move freely. Here, a 10 mm trocar was used for easy access to the gauze.

### Active Peritoneal Inflation or Passive Peritoneal Inflation

#### Actively Break the Peritoneum and Inflate

During the process of freeing the extraperitoneal space, the peritoneum should not be damaged. The extraperitoneal space was inflated perfectly in our operations. Pressing the peritoneum at this time may damage the intestinal tube under the peritoneum. Second, when suturing the fixation patch, because the peritoneum is not damaged and is intact, the peritoneum is tightly attached to the intestinal tube by the pressure of the extraperitoneal space. Suturing the peritoneal fixation patch may cause serious complications such as suturing of the intestine, resulting in an intestinal fistula. At this time, it is necessary to actively break the peritoneum and inflate. Under the navel, a small longitudinal incision is actively applied to the peritoneum. With the help of the pressure from the carbon dioxide pneumoperitoneum, the abdominal cavity is quickly inflated, and a gap is formed directly between the intestine and the peritoneum by the gas. To isolate the gap, the exhaust of the abdominal cavity at the same time, to ensure the operation space of the extraperitoneal space. This way, there is no need to worry about the safety of the lower bowel when suturing the peritoneal fixation patch. The peritoneum is cut longitudinally, standing on the side of the head, and the suture will be smoother and more convenient when closing the peritoneum. It is more horizontal or oblique. The incision for active peritoneal inflation should be lower, that is, below the navel, rather than above. The upper part of the peritoneum should be sutured to close the peritoneum. The two operators must work very hard, and the arms will be framed. This is not conducive to later stitching.

#### Passive Peritoneal Inflation Occurs When the Peritoneum Is Broken During the Freeing Process

**Figure 4:**
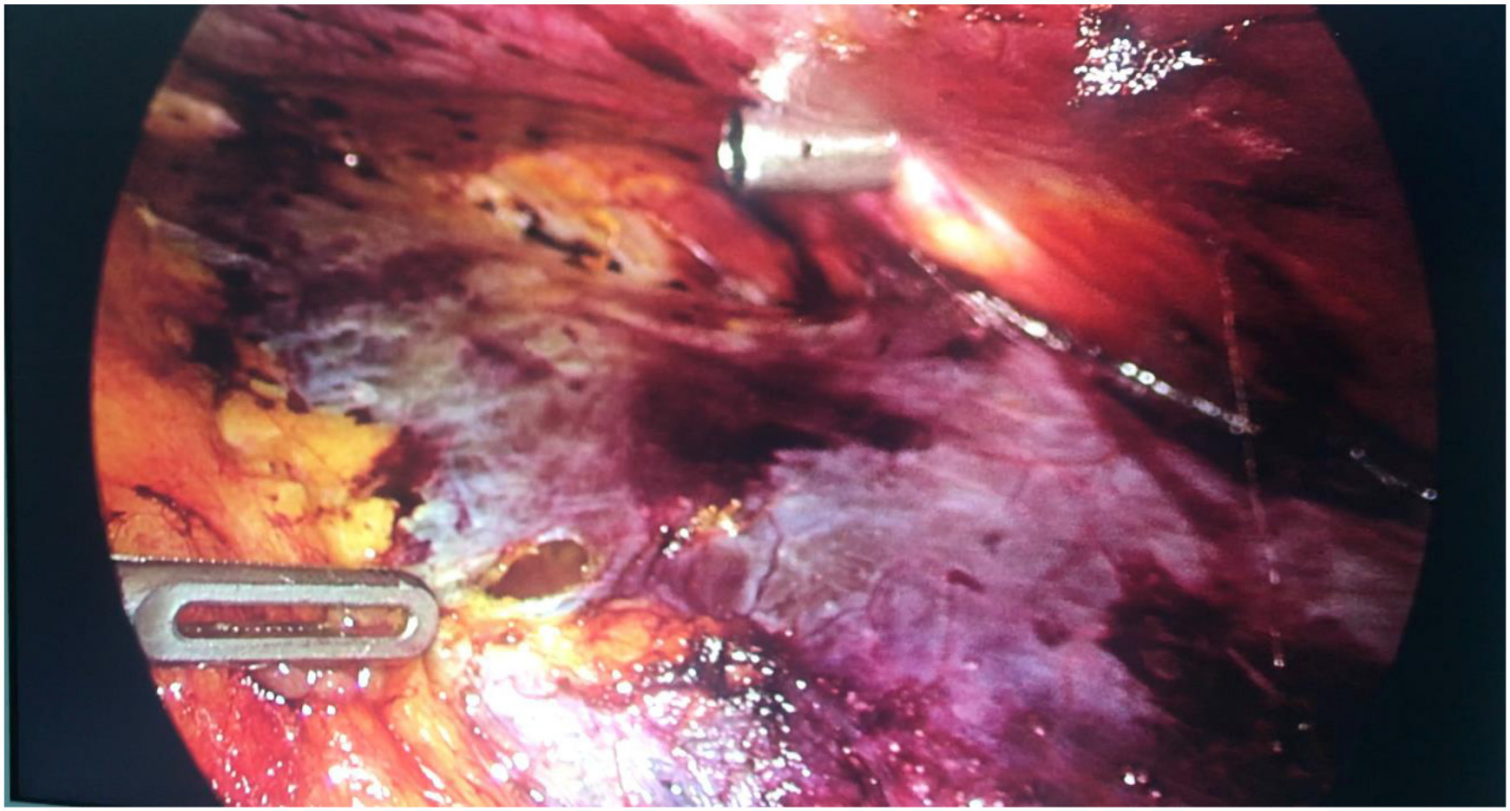
Treatment of peritoneal damage.

At this point, the gas enters the extraperitoneal space naturally, without the need for active re-incision. Then, the abdominal contents form a layer creating the (intestine)-gas-peritoneum-extraperitoneal space (operation space). The peritoneal damage is shown in Figure 4.

### Gauze Ball Blunt Separation Method

**Figure 5:**
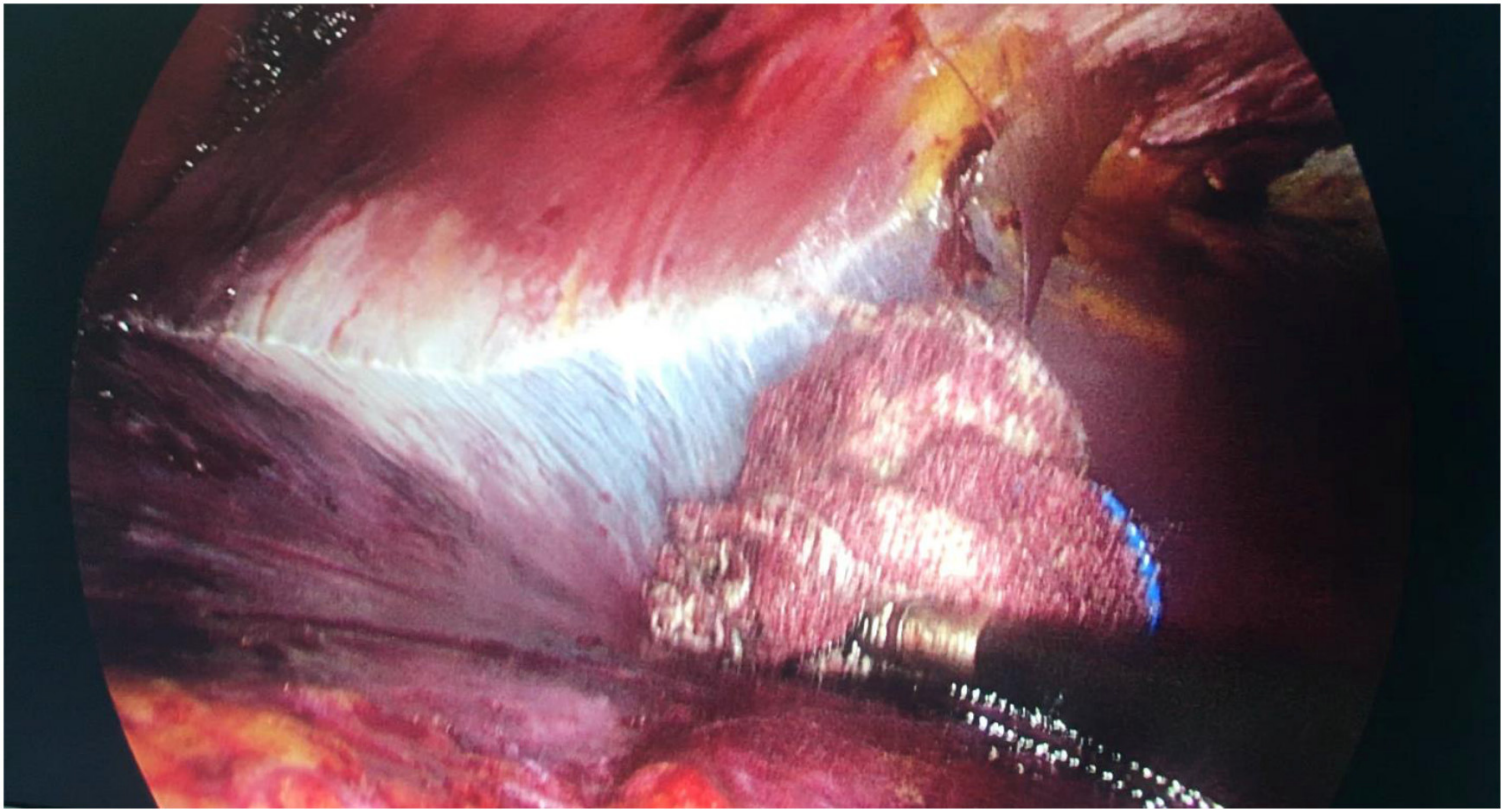
Blunt separation of gauze balls.

When freeing the extraperitoneal space, the steps are as follows: pass through one of the 10 mm puncture holes, enter the laparoscopic gauze, use non-invasive forceps to crimp the gauze, push the abdominal wall upward with the left hand, push the gauze ball forward with the right hand, and press the peritoneum downward. This separation method is very fast and can quickly free the gap, as shown in Figure 5. However, the shortcoming of the slow release of the electric hook is blood oozing, which causes the wound to be unsightly and elderly patients can easily compress the peritoneum. The gauze ball pressure method is based on the action of the abdominal gas.

### Age Factors

For elderly patients over 60 years of age, the peritoneum is thinner, in worse condition, less tough, and less resistant to tension. Compared with young people, there is a greater chance of peritoneal rupture (12). This also leads to many difficulties in applying peritoneal sutures in the later stage. In this case, if you have insufficient experience, a timely transfer of the TES (post-muscle space) or IPOM is a good treatment option.

### Level Selection

The preferred level in which to incise is between the transverse abdominal fascia and the posterior sheath. In the case at the Hunan Provincial People's Hospital, we entered between the peritoneum and transverse abdominal fascia on one side of the operation surface. In theory, this level is the most ideal. There is a layer of peritoneum from the abdominal cavity. However, at this level, the peritoneum is too thin. It is as thin as a cicada's wing and is very easy to break. Therefore, it is not the ideal level in practice. Therefore, the thin transverse abdominal fascia is reserved for the peritoneum, which promotes the peritoneum to become relatively thick, so that the peritoneum is not easily broken during the separation process.

### Peritoneal Rupture Suturing

Peritoneal ruptures near the lower abdomen are easy to suture. Choose round needles with 3–0 absorbable sutures. Paraffin oil lubricates the sutures all the way, so that when the sutures pass through the peritoneum, any trauma to the peritoneum will be minimized (13).

a. If multiple peritoneal breaks are close together, a continuous suturing method should be adopted; that is, after the first break is closed by the suture, do not cut the thread. After tightening, pull the thread over and continue suturing the second break until the continuous suture is completed. If three or four breaches occur, continuous stitching can avoid the cumbersome process of suturing, cutting, ending, and restarting at each breach, enabling the process to be more efficient.

b. If the rupture is large, separate the peritoneum from the front axillary line or even the mid-axillary line on both sides to promote relaxation of the peritoneum. In this way, it will be much easier to close the peritoneal rupture because there will be no tension.

c. If the rupture is large and the peritoneum is free on both sides, and it is still not possible to suture the bulge or the tension after the suture is large, then the greater omentum should be used instead. When this method was employed in our study, the appropriate omentum tissue was selected, and the peritoneal rupture was lifted to the extraperitoneal space. Then the peritoneal rupture was filled, and the greater omentum was continuously sutured along the edge of the peritoneum to fix the peritoneum. In this way, the peritoneal rupture was sealed. The disadvantage of this is that the omentum will adhere to the peritoneum, creating an artificial adhesion, which could potentially lead to internal hernia formation or cause abdominal pain after surgery.

d. Peritoneal rupture can be performed by stamping. Use an anti-adhesion membrane to directly cover the peritoneum to prevent peritoneal rupture. If ruptures are prevented, then stitching is not necessary, thus making the procedure more efficient.

## Results

Twenty-one cases of umbilical hernia repairs in this group were successfully completed. The operation time was 120–240 min (average time, 177.3 ± 42.15 min), and the blood loss volume was 30–40 ml (average, 33.73 ± 3.55 ml). The patch was a Xinhua Shanxiu® Light Hernia Repair Patch. There were 20 cases with a 15 × 15 cm^2^ cut, and 1 case with a Medtronic polypropylene patch and a 15 × 15 cm^2^ cut, and the hernia ring was closed using a hernia needle. The mean follow-up period was 6 months, and there were no short-term complications and no cases of recurrence.

The 21 patients had no postoperative complications and were discharged from the hospital. Since follow-up, there have been no cases of recurrence. The surgeon mainly used the cephalic approach.

We performed an RA separation in a patient. We cut the posterior sheath and peritoneum together, freed the peritoneum, and tried to separate it from the posterior sheath; however, this method failed many times. We therefore had to change the area to the posterior rectus abdominis and operated in the anterior clearance of the posterior sheath. The purpose of the thread that is reserved on both sides is to stretch the left and right sides of front sheath and back sheath incisions, so that the trocar or lens will not enter the wrong level during the later operation as a result from the back sheath incision being pulled up by the thread. In addition, at the end of the operation, it is simple and quick to directly suture the slings on both sides of the carina, which is much faster and safer than suturing alone.is not suspended, the suture when closing the incision after the operation is more laborious and time-consuming. The suturing on both sides of the incision of the anterior sheath and the posterior sheath was performed with a single needle by early surgeons. Because the force area of the anterior and posterior sheath is limited, especially when the posterior sheath is relatively weak, tearing can easily occur, leading to failure of the pulling mechanism. Later, when switched to a continuous suture suspension, the force-bearing area is relatively large, and the aponeurosis is not easy to tear.

Under the cephalic approach, peritoneal ruptures in the upper abdomen are difficult to suture; therefore, care must be taken during the freeing process to prevent peritoneal ruptures. If there is damage, one can lower the patient's head and raise their legs; however, this means that the surgeon's upper limbs are raised during suturing. This will make the surgeon's arms very uncomfortable. Putting a pedal is a good method for the surgeon to improve their comfort.

To date, we have performed a total of 21 cases of umbilical hernia repairs with a cephalic approach plus an extraperitoneal approach on the posterior sheath, and all of them have been successful. A special case was an elderly patient over 60 years of age. Because of the increased age, the toughness of the peritoneum is decreased, so we need to pay more attention when freeing the peritoneum to prevent breaking it. If it is further damaged, there will be further complications during later suturing of the peritoneum.

The advantages of this procedure are that it utilizes the ideal surgical repair level and does not disturb the four-in-one structure of the anterior sheath, rectus abdominis, posterior sheath, and white line. In the extraperitoneal space, the abdominal cavity is not disturbed (14). The patch has the greatest pressure. We believe that it is the perfect method for hernia repair. It is the closest to the abdominal cavity and is worthy of clinical promotion.

### Limitations

This study has some limitations. First, the outcomes of this study are not based on randomized data, and all data were retrospectively collected and analyzed, which creates a potential risk of selection bias. Furthermore, follow-up appointments were made only at a mean of 6 months. Long-term postoperative complications may have occurred thereafter.

## Conclusion

The laparoscopic cephalic approach plus a posterior sheath and extraperitoneal approach is a feasible, safe, and easy-to-incorporate surgical approach. The intraoperative complication rate was low. Prospective research and a larger patient cohort are needed to further confirm this novel surgical technique.

## Data Availability Statement

The raw data supporting the conclusions of this article will be made available by the authors, without undue reservation.

## Ethics Statement

The studies involving human participants were reviewed and approved by The First Affiliated Hospital of Henan University of Traditional Chinese Medicine. The patients/participants provided their written informed consent to participate in this study.

## Author Contributions

All authors contributed to data analysis, drafting or revising the article, gave final approval of the version to be published, and agree to be accountable for all aspects of the work.

## Conflict of Interest

The authors declare that the research was conducted in the absence of any commercial or financial relationships that could be construed as a potential conflict of interest.

## Publisher's Note

All claims expressed in this article are solely those of the authors and do not necessarily represent those of their affiliated organizations, or those of the publisher, the editors and the reviewers. Any product that may be evaluated in this article, or claim that may be made by its manufacturer, is not guaranteed or endorsed by the publisher.
